# A Comparative Overview of Epigenomic Profiling Methods

**DOI:** 10.3389/fcell.2021.714687

**Published:** 2021-07-22

**Authors:** Mahya Mehrmohamadi, Mohammad Hossein Sepehri, Naghme Nazer, Mohammad Reza Norouzi

**Affiliations:** ^1^Department of Biotechnology, College of Science, University of Tehran, Tehran, Iran; ^2^Department of Electrical Engineering, Sharif University of Technology, Tehran, Iran

**Keywords:** epigenomic assays, assay comparison, single-cell epigenome profiling, multi-omics methods, DNA methylation, chromatin profiles

## Abstract

In the past decade, assays that profile different aspects of the epigenome have grown exponentially in number and variation. However, standard guidelines for researchers to choose between available tools depending on their needs are lacking. Here, we introduce a comprehensive collection of the most commonly used bulk and single-cell epigenomic assays and compare and contrast their strengths and weaknesses. We summarize some of the most important technical and experimental parameters that should be considered for making an appropriate decision when designing epigenomic experiments.

## Introduction

**FIGURE 1:**
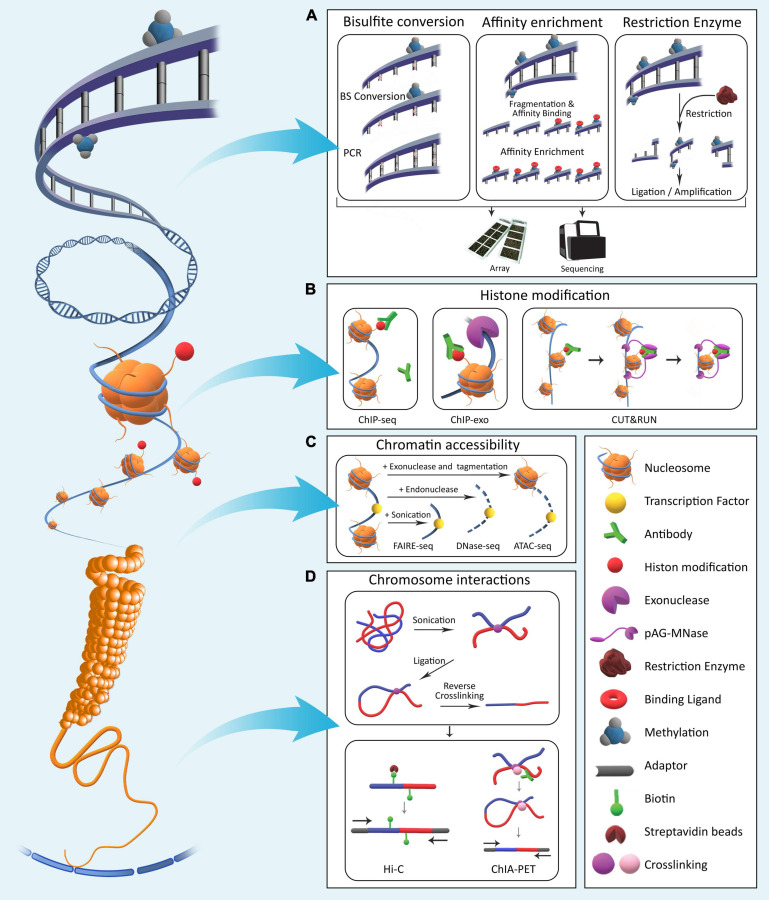
The four major epigenetic layers in Eukaryotic genomes. **(A)** DNA methylation involves direct chemical addition of a methyl group to certain bases in DNA. Methods for assessment of genome-wide DNA methylation are broadly categorized into bisulfite conversion-based, affinity enrichment-based, and restriction enzyme-based techniques. **(B)** Histones undergo a variety of chemical modifications on their tail domains. Methods for detection of these modifications rely on antibodies specifically designed to bind modified histone tails for immunoprecipitation with varying levels of resolution. **(C)** Genomic regions differ with respect to nucleosome occupancy and accessibility of DNA molecule to proteins. Various methods have been developed that quantify these characteristics across the genome. **(D)** Long-range interactions exist between regulatory elements across the genome. To identify and characterize them in a genome-wide fashion, various methods based on crosslinking and ligation have been developed with varying levels of coverage and specificity (Table 1).

Epigenomics involves the profiling and analysis of epigenetic marks across the genome. Epigenetic processes in Eukaryotes generally consist of four major mechanisms: DNA methylation, histone modifications, chromatin compaction, and nuclear organization. These processes modify local genome activity without changing the underlying DNA sequences and thus determine cellular phenotypes by regulating gene expression dynamics (Allis and Jenuwein, 2016). Using epigenetic processes, gene expression programs are inherited through cell generations. Various molecular laboratory techniques have been developed over the years for in-depth studying of the epigenome (DeAngelis et al., 2008; Figure 1).

**TABLE 1:** Comparison of methods for epigenome analysis on bulk samples.

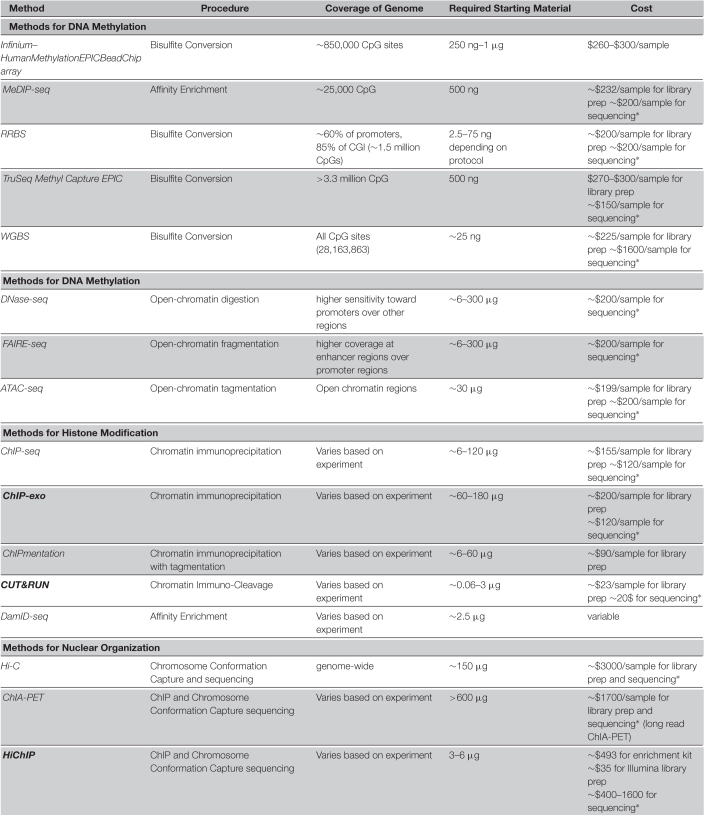

*Sequencing cost estimates are based on Illumina NovaSeq 6000 (SP) 300 Cycle Sequencing.

Some of the most common goals in performing an epigenomic study include: Identification and functional annotation of regulatory elements in the genome; Understanding the dynamics of gene expression regulation in various physiological contexts; Understanding cell-cell heterogeneity and determinants of cell-type specific functions; Characterizing timing of developmental events, gene expression inheritance, and lineage determination; Understanding mechanisms of diseases with epigenetic players; and Drug or biomarker discovery for human conditions.

The diversity of epigenomic assays has expanded in the recent years through both adapting traditional low-throughput techniques, as well as the invention of novel technologies for epigenome-wide high-throughput experiments (Rivera and Ren, 2013; Wang and Chang, 2018). It has thus become increasingly critical to choose the right type of assay depending on the goals, context, and applications in mind. Recently, the number of comparative studies using experimental or simulated data for DNA methylome (Kacmarczyk et al., 2018; Heiss et al., 2020; Tanić et al., 2021) and chromatin accessibility assays (Chang et al., 2018; Gontarz et al., 2020) has increased. However, clear guidelines that integrate the available information to help researchers base their choice among the large selection of epigenomic assays on the most relevant criteria are lacking. In this review, we aim to provide a comprehensive overview of epigenome profiling methods and compare their characteristics that are critical in determining the most appropriate method for a given application.

## Array-Based Assays

Several assays have been developed to measure the epigenome-wide DNA methylation, histone modifications, and chromatin accessibility using hybridization to pre-designed microarrays. Array-based methods for DNA methylation are mainly designed to capture the predominant cytosine modification, 5-methylcytosine (5mC), though certain adaptations have been used to distinguish 5mC from its oxidation derivatives, such as 5-hydroxymethylcytosine (5hmC) (Smith et al., 2019). As the cost per base of sequencing DNA continues to drop, array-based methods are going to become obsolete in the near future. However, given the wealth of the existing DNA methylation array data available for research purposes today, we briefly summarize the basics of these technologies in the following as they also serve as the basis for many newer sequencing-based methods.

### Using Restriction Enzymes

Methylation-sensitive restriction enzymes (MSREs) and methylation-dependent restriction enzymes (MDREs) have been classically used to analyze the local methylation pattern of 5′-Cytosine–phosphate–Guanine-3′ (CpG) dinucleotides in DNA (Oakes et al., 2006). In theory, these methods digest either only the methylated or only the unmethylated fraction of their target sites. Following size selection, fragments of interest can be labeled and hybridized to pre-designed arrays. *Hpa*II tiny-fragment enrichment by ligation-mediated PCR (HELP) is a restriction enzyme-based assay in which the *Hpa*II enzyme (methylation sensitive) and its isoschizomer *Msp*I (methylation insensitive) are used separately to treat DNA (Khulan et al., 2006). Other assays similar to HELP that use comparative approaches between isoschizomers or one MSRE and mock treatment include methylated CpG island (CGI) amplification in combination with microarrays (MCAM) (Estecio et al., 2008), differential methylation hybridization (DMH) (Yan et al., 2009), and comprehensive high-throughput arrays for relative methylation (CHARM) (Irizarry et al., 2008).

### Using Affinity Enrichment

In restriction enzyme-based approaches, only a small portion of the methylome can be assayed. An effective alternative is affinity enrichment of DNA. In this approach, DNA is first sheared randomly either by sonication or enzymatic digestion. Antibodies aimed against methylated CpGs are used to enrich DNA in methylated sequences relative to input control DNA. The resulting enriched fraction and input control are differentially labeled and hybridized to an array platform. In the methylated DNA immunoprecipitation (MeDIP)-chip method, genomic DNA is sonicated to a size range of 300–1000 bp and then subjected to immunoprecipitation (IP) using an anti-5′-methylcytosine monoclonal antibody for comparison with the input DNA. The two samples are differentially labeled and hybridized to a CpG array (Weber et al., 2005; Pelizzola et al., 2008). Methyl binding domain (MBD)-based affinity purification is an alternative approach that enriches hypermethylated DNA fragments (Rauch et al., 2009).

### Using Bisulfite Conversion

Bisulfite treatment is a chemical reaction that converts unmethylated cytosines to uracil by deamination while leaving methylated cytosines unconverted (Clark et al., 1994, 2006). Bisulfite conversion-based approaches offer single CpG resolution and are considered the gold-standard technique for DNA methylation assessment as discussed in more detail in the following sections. However, coupling bisulfite treatment with array hybridization has been challenging due to the lower sequence complexity of the converted DNA. The Illumina GoldenGate BeadArray and Infinium arrays are commercially available arrays specifically designed and optimized for this purpose. The most recent Infinium HumanMethylation BeadChip, HumanMethylationEPIC, measures approximately 850,000 cytosines across the genome.

## Sequencing-Based Assays

### Using Restriction Enzymes

Restriction endonuclease enzymes have been combined with sequencing to reduce the total costs of sequencing-based methylome assays. For example, the original HELP assay has been improved and coupled with next generation sequencing (NGS) (Oda et al., 2009). Using two sets of adaptors to amplify fragments smaller than 200 base pairs (bp) during the ligation mediated PCR step and coupling HELP output with NGS, it is possible to analyze 98.5% CGIs in the human genome (Oda et al., 2009).

### Using Bisulfite Conversion

Bisulfite sequencing (BS-seq) involves converting all unmethylated cytosines in the DNA sample to uracil by deamination, with no effect on methylated cytosines, followed by sequencing (Hayatsu, 2008). BS-seq is currently considered the gold-standard single base-resolution assay for DNA methylation. However, methods that include bisulfite conversion generally suffer from the following issues: Reduced sequence complexity due to the conversion of unmethylated cytosines to thymidine which results in more difficult read alignment; Missing variations such as single nucleotide polymorphisms (SNPs) where a cytosine is converted to a thymidine; Inability to distinguish between 5mC and 5hmC; and partial digestion of DNA during bisulfite treatment. Following bisulfite treatment, if sequencing is performed on the entire DNA sample, it is referred to as whole-genome bisulfite sequencing (WGBS). However, to increase local sequencing coverage and reduce NGS costs, sometimes sequencing is only performed on specific target DNA regions enriched using various methods. One such method is reduced representation bisulfite sequencing (RRBS) that enriches CpG-rich regions non-specifically (Meissner et al., 2005). In this method, restriction digestion is combined with bisulfite conversion and size selection to enrich ∼1–5% of the genome with high CpG density. A more flexible but also more costly alternative is targeted sequencing of enriched genomic regions of interest using custom bisulfite padlock probes (BSPP) (Diep et al., 2012) or hybridization capture probes such as Illumina’s TruSeq Methyl Capture EPIC. The targeted panel covers approximately 3.34 million CpG sites. TruSeq EPIC has a significant improvement over EPIC-array regarding genomic resolution and number of CpGs, however it suffers from lower precision due to limited coverage per site for comparable costs with arrays (Heiss et al., 2020). Other commercially available targeted bisulfite sequencing platforms also exist with comparable characteristics with the TruSeq (Tanić et al., 2021). The significant limitations of methods based on bisulfite conversion remain cost, incomplete conversion, as well as partial DNA digestion during bisulfite treatment. However, much optimization and improvement have been made in recent years to reduce these issues, especially for treatment of low-input DNA samples such as cell-free DNA (Worm Orntoft et al., 2017).

In addition to 5mC, other less common cytosine modifications have been discovered to play important roles in contexts of development and diseases (Sun et al., 2014). These include 5-formylcytosine (5fC), 5-carboxylcytosine (5caC), and 5hmC, that are all oxidation derivatives of 5mC and intermediates in its demethylation. It is important to note that unlike 5fC and 5caC, 5mC and 5hmC are both resistant to bisulfite conversion and therefore cannot be distinguished from each other by standard BS-seq. Some adaptations of the BS-seq protocol that allow discrimination between 5mC and 5hmC have been developed. In oxidative BS-seq (OxBS-seq) (Booth et al., 2012), first 5hmC in genomic DNA is oxidized to 5fC. Unlike 5mC and 5hmC, 5fC is sensitive to deamination by bisulfite; therefore, bisulfite treatment of the oxidized DNA converts the 5fCs to uracil. A standard bisulfite procedure is performed in parallel for identifying both 5mC and 5hmC. By comparing these two sequencing results, 5hmC presence can be inferred. Another discriminative approach between 5mC and 5hmC is Ten-eleven Translocation (Tet) assisted bisulfite sequencing (TAB-seq) (Yu et al., 2012) in which 5hmC is first glycosylated and thus protected from oxidation by Tet1. This allows subsequent oxidation with the Tet enzymes of 5mC followed by bisulfite treatment, where only 5hmC remains unchanged. The significant advantage of OxBS-seq over enzymatic approaches such as TAB-seq is that OxBS-seq does not require highly active Tet enzymes which can be expensive and are only about 95% efficient. The major disadvantage of OxBS-seq is that two sequencing runs are performed and subtracted, thus error rate and cost are increased. Another method in this family which makes use of restriction endonuclease coupled with DNA glycosylation is AbaSI endonuclease digestion coupled with sequencing (Aba-seq) (Sun et al., 2013). Finally, h-MeDIP-seq uses immunoprecipitation of DNA fragments containing 5hmC, followed by sequencing (Tan et al., 2013).

### Using Direct Detection During Sequencing

The development of long-read sequencing by the Pacific Biosciences (PacBio) platform allows for detecting DNA modifications including cytosine and the less well characterized N6-adenine methylation (Xiao et al., 2018), directly during sequencing (Yang and Scott, 2017). Single-Molecule Real-Time (SMRT) sequencing enables the analysis of real-time DNA polymerase kinetics for inference of DNA base modifications (Tse et al., 2021). This technology can detect DNA polymerase kinetics changes due to base modifications such as methylation (Flusberg et al., 2010). Another unique sequencing technology with the power of discriminating methylated and unmethylated cytosines is the nanopore sequencing (Simpson et al., 2017). This sequencer generates electrical signal that can be analyzed to determine methylation states without any chemical treatments. The main advantage of these methods is that they obviate the need for high levels of input DNA unlike bisulfite-based alternatives.

### Using Affinity Enrichment

Another alternative to bisulfite conversion-based determination of DNA methylation state is protein facilitated enrichment. Enrichment of methylated DNA is possible with anti-methylcytosine binding proteins (MBD) or antibodies against 5mC (MeDIP), followed by sequencing. Alignment of reads elucidates peak regions with higher DNA methylation than the genome-wide background. This method’s main limitation is coverage of only methylated sites and imperfect specificity of antibodies. MBD methods [like MIRA-seq (Jung et al., 2015)] are more powerful in enrichment of CGIs, while MeDIP-seq better enriches regions with low CpG density (Harris et al., 2010). Both affinity enrichment methods lack single-base resolution and are sensitive to CpG density, fragment size, and efficiency of enrichment.

Affinity-based methods are also highly popular for assaying layers of the epigenome other than the DNA methylome. A popular technique used to analyze DNA-protein interactions including histone modifications relies on chromatin immunoprecipitation (ChIP) (Milne et al., 2009). This method takes advantage of antibodies with specific binding affinity to histone modification of interests. The pull-down of chromatin fragments attached to these antibodies allows for the separation of genomic regions harboring the mark from those lacking it. The primary method for epigenome-wide assessment of histone marks is ChIP followed by sequencing (ChIP-seq). As the name suggests, this method relies on the vast number of histone-mark-specific commercially available antibodies. Determining the positions of peaks in sequencing coverage informs of the epigenetic state of the underlying genomic position. ChIP-exo is a modification of ChIP-seq that allows higher resolution of binding sites, from hundreds of base pairs in ChIP-seq to a single base resolution. It takes advantage of exonucleases to digest the protein-bound DNA upto a few base pairs that are directly bound to the histone modification of interest (Rhee and Pugh, 2012). ChIP-nexus, is an improved version of ChIP-exo that incorporates a more efficient library preparation method through intramolecular ligation, though it is more costly (He et al., 2015). Finally, ChIPmentation (Schmidl et al., 2015) is a technique that performs tagmentation with Tn5 transposase directly on ChIP fragments, followed by sequencing, lowering cost and input requirements of standard ChIP-seq.

An alternative method for histone profiling that has recently gained popularity due to lower input requirements is Cleavage Under Targets & Release Using Nuclease (CUT&RUN) (Skene and Henikoff, 2017). This method, starts by targeting DNA-bound protein of interest in isolated nuclei using specific antibodies, followed by treatment with micrococcal nuclease conjugated with protein A (MNase-pA). After attachment of the antibody to the target protein in intact cells, MNase cuts off the DNA to which the protein is bound, releasing these short DNA fragments for subsequent sequencing. Cleavage Under Targets and Tagmentation (CUT&Tag) addresses some of the shortcomings of CUT&RUN, such as DNA loss owing to MNase fragmentation, through utilization of a Tn5 transposase-Protein A (pA-Tn5) fusion protein loaded with sequencing adapters (Kaya-Okur et al., 2019).

IP-based methods are limited by reliance on antibodies. An alternative is a technique based on the expression of a fusion protein consisting of the *Escherichia coli* deoxyadenosine methylase (Dam) and the protein of interest (in this case, histone readers or modifiers) called DamID (Greil et al., 2006). This allows Dam to methylate DNA on adenine residues in GATC sequences close to the protein of interest’s binding sites, therefore it is limited by GATC sequence occurrences. Following methylation, enrichment by restriction digestion and sequencing are performed using various protocols (Aughey et al., 2019). The main limitations of DamID based methods are the need for transgenic cells and high background due to off-target methylation.

### Using Open-Chromatin Digestion

Another family of epigenomic experiments focus on determining the compactness of different genomic regions, regardless of the chemical nature of epigenetic marks. These approaches quantify nucleosome positioning and chromatin accessibility using various molecular techniques. The first of these methods to be developed were DNase I hyper-sensitive sites sequencing (DNase-seq) (Boyle et al., 2008; Song and Crawford, 2010) and micrococcal nuclease digestion with deep sequencing (MNase-seq) (Schones et al., 2008). Both of these methods make use of endonuclease enzymes to fragment DNA in regions not occupied by histones and other proteins. Following size selection and sequencing of fragmented DNA, nucleosome positions are inferred. DNase-seq mainly enriches open chromatin regions that exhibit hypersensitivity to degradation by DNase I, thus directly determining regulatory regions (promoter and enhancers), while MNase-seq allows indirect inference of gene regulatory regions from nucleosome occupancy. Formaldehyde-assisted identification of regulatory elements followed by sequencing (FAIRE-seq) is another similar assay that takes advantage of the fact that genomic DNA within open chromatin regions is particularly sensitive to shearing by sonication (Giresi et al., 2007). Assay for Transposase-accessible chromatin using sequencing (ATAC-seq) is the most recent chromatin accessibility assay developed. This method uses the Tn5 transposase in the NGS library preparation step (Buenrostro et al., 2015a). A hyperactive mutant Tn5 transposase inserts sequencing adapters into open regions of the genome (tagmentation). The tagged DNA fragments are then purified, PCR-amplified, and sequenced by NGS. The use of tagmentation largely reduces the number of input DNA needed and makes ATAC-seq the fastest and most sensitive of the available assays. It is important to note that the ATAC-seq method is associated with some technical limitations that can introduce bias. For example, some bound chromatin regions might open and become tagged during sample processing. Furthermore, fragments tagged by adaptors without the correct orientation and appropriate spacing required for amplification are lost during PCR. Potentially high proportions of mitochondrial fragments due to lack of chromatin packaging and variable nuclei preparation qualities are among other confounding factors for an ATAC-seq library (Orchard et al., 2020). Nonetheless, the low input material requirement, *in situ* library preparation, and time efficiency of ATAC-seq make it the current gold-standard in the field (Table 1).

### Using Chromatin Conformation Capture

Distant chromatin regions from different chromosomes interact with one another, and this three-dimensional nuclear organization is important for all nuclear processes, including replication, repair, and gene expression (van Steensel and Belmont, 2017). Nuclear territories including lamina associated domains (LAD) and topology associated domains (TAD) allow for spatial regulation of gene expression. Detailed characterization of the boundaries of these domains and long-range chromosome interactions within them is possible through chromatin conformation capture (3C)-based methods. These methods rely on cross-linking and ligating physically interacting chromosomal regions. Proteins are first cross-linked to associated DNA and to themselves followed by restriction digestion. This generates fragments, including cross-linked genomic regions close together in the three-dimensional nuclear space but on different chromosomes or thousands of base pairs away in the linear genome sequence. Next, proximity ligation is used to connect the ends of DNA fragments previously cross-linked through their associated proteins. Next, the cross-linking is reversed, resulting in linear DNA fragments. Various methods can then be applied downstream for characterization of interacting domains from different chromosomes. Hi-C is the genome-wide version of 3C that uses NGS for high-throughput quantification of all chromatin interactions (van Berkum et al., 2010). Ligation products between distant regions are enriched and then subject to deep paired-end sequencing. This generates sequencing reads from both ends of fragments, and for ligated fragments, the two reads map to different regions in the genome (Figure 1). Methods such as chromatin interaction analysis by paired-end tag sequencing (ChIA-PET) (Fullwood et al., 2009) and HiChIP (Mumbach et al., 2016) combine HiC with ChIP. These methods are thus protein centric and start by pulling down chromatin associated with a protein of interest. HiChIP methods such as proximity ligation-assisted ChIP-seq (PLAC-seq) (Fang et al., 2016) achieve higher accuracy and efficiency compared with ChIA-PET by performing cross-linking, restriction digest, biotinylation, and ligation steps all within the nucleus, prior to sonication and immunoprecipitation.

## Multi-Omics Assays

Studying each of the layers in the epigenome, namely the DNA methylome, histone marks, chromatin accessibility, and interaction domains separately, provides independent information regarding the dynamics and variation of gene expression by each of these processes. However, direct inference of function or phenotype from these data proves to be complicated. For instance, an increase in DNA methylation can lead to over- or under-expression of corresponding genes depending on the chromatin context (Wagner et al., 2014). Therefore, integrating multiple layers of epigenomic data harboring complementary information has been insightful in understanding chromatin function and gene expression regulation.

Several methods have been developed for simultaneous profiling of multiple epigenomic layers across the genome in recent years. For instance, nucleosome occupancy methylome sequencing (NOME-seq) can measure DNA methylation and chromatin accessibility in the same sample at once (Kelly et al., 2012). This approach takes advantage of the methyltransferase enzyme M.CviPI that methylates 5′-Guanine–phosphate–Cytosine-3′ (GpC) sites in nucleosomes-free regions. Followed by bisulfite conversion and whole-genome sequencing, the results can then provide a footprint of nucleosome positioning and DNA methylation in one assay. Another method named EpiMethylTag combines bisulfite conversion with ATAC-seq or ChIP-seq (M-ATAC or M-ChIP, respectively) (Lhoumaud et al., 2019). This allows for simultaneous examination of methylation and accessibility/histone modification on the same DNA molecules. ATAC-Me, is another method similar to EpiMethylTag that combines ATAC-seq and BS-seq (Barnett et al., 2020). Compared with NOMe-seq, ATAC-based methods are not limited by the GpC dinucleotide frequency of loci (Klemm et al., 2019). A wide range of such integrative multi-level epigenome-wide assays have been introduced, and rapid advancements are being made in optimizing them in terms of costs, required input DNA, coverage representation among other factors.

Multi-omic assays are attractive in general for elucidating the dynamics and interactions between molecular mechanisms in the cell. Particularly, the field of epigenomics can benefit from such studies since the relationship between various layers of the epigenome are not fully characterized and understood yet. The downside remains lower throughput of multi-omic methods compared to single assays. Therefore, for applications where high quality and high depth data on a particular epigenomic layer is required, single-omic assays still take priority.

## Single-Cell Assays

**FIGURE 2:**
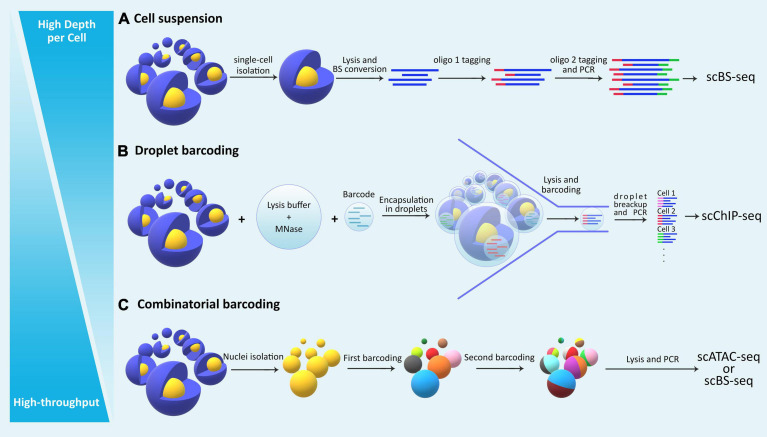
The general categorization of single cell epigenomic assays. **(A)** Methods that start by dissociating cells in a sample into a single-cell suspension followed by lysis of individual cells in physically separated compartments/reactions, reach high depth of information per cell, but are laborious and expensive and thus offer limited throughput. **(B)** Droplet based methods allow single-cell identification using barcoding without the need for physical compartmentalization of individual cells, and thus reduce cost and increase throughput. **(C)** Methods that use combinatorial barcoding on isolated nuclei offer many advantages including higher throughput, albeit in the expense of depth of information per individual cell.

Single-cell (sc-) omics assays have advanced significantly in the past decade, empowering researchers to tackle problems such as cell-cell heterogeneity and rare cell population characterization. Studying the epigenome in such contexts with classical bulk methods is not feasible due to lack of resolution and the requirement for high starting material. In recent years, researchers have taken advantage of advancements in single-cell sequencing technologies toward developing high-resolution epigenomic assays at the level of individual cells (Lo and Zhou, 2018). Integrative multi-omic approaches at the single-cell level have also been developed for multiple layers of the epigenome that provide functional insights about the interaction between them in a given cell (Kelsey et al., 2017). The main challenges with sc-epigenomic assays remain high variability, low coverage per cell, limited throughput (total number of cells analyzed from a sample), and high costs (Table 2). Among different single-cell epigenomic approaches, the choice mainly depends on two parameters: throughput (total number of cells) and depth per cell (Figure 2).

**TABLE 2:** Comparison of single-cell epigenomic methods.

**Method**	**Advantages**	**Disadvantages**
**Methods for Single-cell DNA Methylation**		
***scRBBS-seq***	High coverage of the CpG islands	Low coverage of genome-wide sparse CpGs
***scPBAT***	High genome-wide CpG coverage; Low input requirement	Adaptor ligation bias
***scCGI-seq***	High coverage and consistency in CpG islands profiling	Low coverage of genome-wide sparse CpGs, Low-throughput
***sci-MET***	High-throughput and high alignment efficiency	Low coverage per cell
**Methods for Single-cell Chromatin Accessibility**		
***sci-ATAC-seq***	High-throughput	Low coverage per cell
***scATAC-seq (droplet-based)***	High coverage of reads (in comparison with sci-ATAC-seq)	Low-throughput
***scDNase-seq***	High-sequencing resolution	Low throughput and mapping efficiency
**Methods for Single-cell Histone Modifications**		
***scCHIP-seq***	High-throughput	Low coverage per cell
***scDam-ID***	No target-specific antibody required, suitable for identification of loose or indirect associations	Resolution limited by the frequency of methylation sites
***scChIC-seq***	High number of obtained unique reads per cell	Low throughput
***scCUT&TAG***	Cost-effective, High-throughput	Low number of unique reads
***CoBATCH***	High throughput, High number of obtained unique reads with low background	Unsuitable for repressive marks detection; non-specific cleavage of accessible regions
***ACT-seq***	High throughput, Simple workflow	Low number of unique reads; non-specific cleavage of accessible regions
***scCHIL-seq***	Low background	Time-consuming and complex workflow
**Methods for Single-cell Nuclear Organization**		
***scHi-C***	High coverage per cell	Low-throughput
***sciHi-C***	High-throughput	Low depth per cell
**Methods for Single-cell Multi-omics**		
***scM&T-seq***	High genome-wide CpG coverage	Medium throughput
***scMT-seq***	High coverage of the CpG islands	Low throughput, Low coverage of genome-wide sparse CpGs
***scCOOL-seq***	High coverage of the CpG islands and promoter regions	Low GCH coverage, high sequencing depth needed
***iscCOOL-seq***	Improved throughput and mapping efficiency	Low GCH coverage, high sequencing depth needed
***scNMT-seq***	Medium throughput	Low coverage of genome-wide CpGs
***Methyl-HiC***	Able to identify cell-type specific chromatin interactions	Low-throughput
***sn-m3C-seq***	Higher mapping efficiency than scNMT; cell-cell differences in chromatin conformation are hard to detect	Low throughput

Single-cell epigenomic assays can be broadly divided into three categories (Figure 2): methods that isolate and compartmentalize single cells using various techniques reviewed previously (Hu P. et al., 2016), methods that use droplet barcoding (Rotem et al., 2015), and methods that use combinatorial barcoding on single-nuclei (Cusanovich et al., 2015). In general, methods that lyse single cells require dissociation of tissues into suspensions, have higher noise levels due to single cell amplification bias, and are more costly and labor intensive, thus offering lower throughput but higher depth per cell. Droplet barcoding methods offer improvements regarding bias, throughput, and cost through performing the single-cell lysis in individual droplets in one step rather than separate reaction volumes. Finally, combinatorial barcoding offers even higher throughput by using barcode combinations, and also bypasses the cell suspension requirement by working on isolated nuclei which are readily obtainable even from preserved tissue samples and tissue that are generally difficult to dissociate. In the following, we will compare some of the established methods and provide an overview of their advantages and disadvantages (Table 2).

### Single-Cell DNA Methylation Assays

Single-cell assessment of the epigenome became first and most readily available for DNA methylation as the closest to the previously established sc-genomic assays and the most convenient epigenomic layer to evaluate using bisulfite conversion. However, BS-seq at single-cell level requires methods for library preparation to mitigate the issue of DNA loss during bisulfite treatment. Sc-RRBS employs sequence-specific fragmentation on genomic DNA using one or more restriction enzymes followed by bisulfite sequencing (Guo et al., 2013). To prevent DNA loss, all DNA processing steps prior to PCR amplification occur in a single tube. Alternatively, post-bisulfite adaptor tagging (PBAT) avoids the bisulfite-induced loss of sequencing libraries by performing adapter tagging and two rounds of random primer extension after bisulfite treatment (Miura et al., 2012). In scBS-seq, a protocol similar to PBAT is used in which after cell separation and lysis, bisulfite treatment is done to simultaneously fragment and convert cytosines in cellular DNA, followed by random priming and extension (Smallwood et al., 2014).

Recent advances have facilitated high-throughput methylome profiling in single cells by combinatorial indexing. Single-cell combinatorial indexing for methylation analysis (sci-MET) employs fluorescence-activated nuclei sorting (FANS) for nuclei isolation and Tn5 tagmentation and PCR for combinatorial indexing of single-cells, followed by NGS (Mulqueen et al., 2018). Single-cell CGI methylation sequencing (scCGI-seq) is a novel bisulfite-independent approach that incorporates MSRE digestion and multiple displacement amplification (MDA) for methyl-CGI detection (Han et al., 2017). MDA is a commonly used isothermal whole-genome amplification method that reduces the PCR-associated amplification bias (Dean et al., 2002). ScCGI-seq has been shown to obtain a high single-cell coverage (>70% of all CGIs) (Han et al., 2017). A number of single-cell methylation assays have also been developed with the ability of distinguishing common derivatives of 5mC such as 5fC and 5hmC (Zhu et al., 2017).

### Single-Cell Histone Modifications Assays

Two primary methods for assessing histone modifications in single cells exist: Drop-ChIP/scChIP-seq (Rotem et al., 2015) and scDamID (Kind et al., 2015). In scChIP-seq, single-cells are first separated into droplets containing lysis buffer and micrococcal nuclease (MNase) and then barcoded prior to the immunoprecipitation step. This increases the efficiency of the pull-down step and therefore reduces the background noise. Single-cell DNA adenine methyltransferase identification (scDamID) is designed to map DNA-protein associations using DamID in single-cells isolated by fluorescence-activated cell sorting (FACS) (de Luca and Kind, 2021).

The CUT&RUN methodology has also been adapted for histone profiling by single-cell chromatin immune-cleavage sequencing technique (scChIC-seq) (Ku et al., 2019; Bartosovic et al., 2021). In scChIC-seq, both target and non-target DNA fragments are retrieved and ligated to adaptors to reduce DNA loss during library construction, but target fragments are selectively amplified during subsequent PCR. Also, Similar to CUT&TAG (Kaya-Okur et al., 2019), several other antibody-directed transposase-mediated methods such as combinatorial barcoding and targeted chromatin release (COBATCH) (Wang et al., 2019), antibody-guided chromatin tagmentation sequencing (ACT-seq) (Carter et al., 2019), and single-cell chromatin integration labeling sequencing (scChIL-seq) (Harada et al., 2019) have been developed to profile protein–DNA interactions across the genome in single cells.

### Single-Cell Chromatin Accessibility Assays

Both DNase-seq and MNase-seq have been modified to allow single-cell resolution profiling on FACS-isolated cells, namely scDNase-seq (Jin et al., 2015) and scMNase-seq (Small et al., 2014).

Single-cell ATAC-seq has also been developed using two approaches: cell-isolation based (Buenrostro et al., 2015b) and combinatorial indexing based (Cusanovich et al., 2015). These methods are compared in Table 2.

### Single-Cell Nuclear Organization Assays

Conventional Hi-C protocol has been modified to create single-cell Hi-C (scHi-C), a technique that can determine chromosomal conformation in individual nuclei (Nagano et al., 2013). In contrast to bulk Hi-C protocol, the ligation step is performed in nuclei before nuclear lysis, followed by single-cell isolation. By combining combinatorial cellular indexing with scHi-C, a method named single-cell combinatorial indexed Hi-C (sciHi-C) has been introduced to determine genome-wide chromatin interaction in tens of thousands of individual cells (Ramani et al., 2017).

### Single-Cell Multi-Omics Approaches

Multi-omics technologies quantify several types of molecules on the same molecule and allow for a multi-layer exploration of cell identity (Zhu et al., 2020). Many of them have been adapted to single-cell resolution. Various methods simultaneously measure DNA methylome and the transcriptome (Angermueller et al., 2016; Hou et al., 2016; Hu Y. et al., 2016; Hu et al., 2019), chromatin accessibility and the transcriptome (Cao et al., 2018; Liu et al., 2019; Zhu et al., 2019), or chromatin accessibility, the transcriptome, and cell surface epitopes (Swanson et al., 2021) at single-cell resolution, which are rapidly advancing in scope and performance. Here, we only focus on techniques for simultaneous measurement of multiple epigenomic layers and provide an overview of them.

The NOMe-seq method has been adapted to single-cell level to determine the chromatin accessibility and DNA methylome of single cells in heterogeneous populations (Pott, 2017). A similar technique called single-cell chromatin overall omic-scale landscape sequencing (scCOOL-seq) was proposed for the simultaneous and parallel measurement of chromatin accessibility, DNA methylation, and copy number variation in single cells by combining scBS-seq with scNOMe-seq (Guo et al., 2017). Recently, improved-scCOOL-seq (iscCOOL-seq) technique has been proposed, which employs single-cell tailing and ligation-free (TAILS) approach to simplify methylome library preparation and increase the mapping efficiency from 22 to 62% (Gu et al., 2019).

Single-cell nucleosome, methylation, and transcription sequencing (scNMT-seq) enables the parallel profiling of chromatin accessibility, DNA methylome, and transcriptome by incorporating scM&T-seq (Smart-seq2 and scBS-seq) and scNOMe-seq (Clark et al., 2018). Methyl-HiC allows for simultaneous profiling of the chromosome conformation and DNA methylome in single cells by performing *in situ* HiC followed by BS-seq (Li et al., 2019). Recently, single-nucleus methyl-3C sequencing (sn-m3C-seq) has been developed to capture chromatin conformation and methylome from the same cell. To perform sn-m3C-seq, a standard *in situ* 3C experiment is first carried out, followed by nuclei sorting, bisulfite conversion, and sequencing (Lee et al., 2019).

### Spatial Epigenomic Approaches

All of the techniques discussed above start by dissociating tissue samples and therefore losing information about the spatial organization of single cells within tissues *in vivo*. Although these assays are currently the most widely used in the field, an independent category of methods under spatial assays that preserve the cell-cell organization in original tissue contexts have emerged and gained popularity (Stahl et al., 2016; Larsson et al., 2021). In this category, epigenome-wide profiling of histone modifications in tissues has recently been proposed and named high-spatial-resolution chromatin modification state profiling by sequencing (hsrChST-seq) (Deng et al., 2021). By combining in-tissue CUT&TAG with combinatorial barcoding and fluorescence imaging, this NGS-based technique offers the first spatially resolved single-cell epigenome profiles which shed new insights onto tissue function and organization.

## Conclusion

The activity of different regions of the genome is fine-tuned through epigenetic processes. This is achieved through superimposed layers of chromatin organization such as DNA methylation, chromatin accessibility, histone modifications, and nuclear organization. In this section, we provide a comparative guide for assay selection among the vast number of epigenomic assays developed to date (Table 1).

### For DNA Methylation Analysis

The main parameters affecting a reasonable choice among the existing methylome assays include assay requirements and convenience, coverage of the genome, resolution, amount and quality of the required starting material, time to results, and throughput which together determine the feasibility and costs associated with different approaches. Affinity enrichment techniques like MeDIP and MBD based methods have the advantage of lower cost and are less labor intensive in comparison to bisulfite conversion based assays, but have lower resolution as they cannot capture DNA methylation at single nucleotide (Singer, 2019). The choice between variations of bisulfite treatment-based methods is mainly dependent on the biological purpose of the assay. While WGBS is the most informative and comprehensive method, it is most suitable for discovery and annotation purposes while being cost-prohibitive for high-throughput experiments (large sample size). In many contexts such as differential methylation analysis for disease biomarker selection, it is not necessary to cover the entire genome and one can focus on CpG-rich regulatory elements where methylation markers are more likely to be located. Alternatively, targeted techniques like methylation arrays and targeted bisulfite sequencing are popular as they reduce costs and increase throughput significantly. The Infinium array is more affordable with simpler lab protocols and data analysis, however RRBS is preferred when there is interest in CpGs outside of the Infinium target list. Targeted techniques also allow for multiplexing of samples for NGS by reducing library size in comparison to WGBS. This also helps decrease batch effect and increase statistical power in high-throughput experiments. While RRBS is less costly than target enrichment methods using customizable probes, it lacks their specificity and flexibility in the choice of target regions. Finally, in contexts such as developmental studies where there is interest in distinguishing 5mC from its oxidation derivatives, assays such as OxBS-seq and Tet-assisted bisulfite sequencing (TAB-seq) are the methods of choice.

### For Chromatin Accessibility

Due to the nature of DNase-seq and FAIRE-seq, they preferentially cover promoters and enhancers, respectively. Generally, of the three main assays for capturing chromatin accessibility, ATAC-seq is the most accurate, offers a protocol that is fast and simple, and requires lower number of input cells (thousands in comparison to millions). Furthermore, it is a versatile and flexible method since it does not require sonication, size selection, phenol-chloroform extraction, or antibodies and thus can be adapted to various research questions. ATAC-seq offers similar sensitivity and specificity when compared to DNase-seq, and superior performance characteristics compared to FAIRE-seq and therefore it has become the preferred method of choice for chromatin accessibility assays.

### For Histone Modifications

Most approaches for the detection of histone modifications at the epigenome level rely on immunoprecipitation. ChIP-seq is a popular method for detection of histone modifications which despite higher cost outperforms the previously common technique, ChIP-chip, by providing better single base resolution, more signal to noise ratio, and narrow peak detection. However, ChIP-seq has its own limitations (Park, 2009). It needs a large amount of starting material and the sensitivity is dependent on the quality of the antibodies. A newer version named ChIPmentation, uses tagmentation (as in ATAC-seq) which reduces the required amount of cells by a factor of 100. CUT&TAG is an antibody-based alternative that obviates the need for high levels of input material. DamID-chip and DamID-seq are alternatives that do not require antibodies or high input DNA (Wu et al., 2016), but the limitation is the need for recombinant fusion proteins and therefore DamID-based methods are still less widely used compared to ChIP-based methods (Table 1).

### For Nuclear Organization

The main assays for nuclear organization analysis and Hi-C and ChIA-PET. Hi-C has the advantage of genome-wide profiling while losing the sensitivity of capturing details of specific protein interactions. To capture this information Hi-C must be performed with very high sequencing depth which is cost-prohibitive. ChIA-PET on the other hand, provides a protein-centric view so one must start with a protein of interest such as a specific transcription factor, and obtain the data regarding its interactions throughout the genome. However, ChIA-PET suffers from low sensitivity. These approaches are compared in Table 1.

### Discussion

The expansion in diversity of epigenomic assays has provided researchers with an unprecedent suite of methods for various applications. These epigenomic assays vary in terms of resolution, cost and availability of equipment and reagents, coverage, and data analysis. The large selection makes it ever more challenging to make the optimal choice for a study. Thus, standard guidelines are required to aid researchers in choosing the best practices depending on the context of their research and their access to equipment. Here, we tried to take one step toward this goal by focusing on some of the most popular epigenomic assays currently in use and providing comparative assessment of their characteristics. However, a very important aspect of this comparison that we did not cover here is the data analysis stage. As different epigenomic assays produce incredibly different types and volumes of data, analysis pipelines and their challenges vary significantly between them.

For the development of reliable best practices guidelines, direct and side-by-side comparisons of epigenomic assays, such as previous comparative studies (Harris et al., 2010; Chang et al., 2018; Kacmarczyk et al., 2018; Gontarz et al., 2020; Heiss et al., 2020; Tanić et al., 2021) are required. Additional analyses are called for to clearly elucidate advantages and disadvantages of each of the methods in a comprehensive manner, perhaps in community efforts in the future. Especially with the rapid expansion of single-cell and multi-omic assays, careful consideration of the most informative and accurate method of choice for a given application has become a difficult task.

Single-cell assays are becoming the routine in many areas where there is interest in the epigenome. The cell-level resolution of these approaches offers not only new insights about cell-cell interactions, cellular heterogeneity, and cellular sub-populations, but also provides a means for studying rare cell populations such as early stages in development. Single-cell assays can also prove useful in contexts where starting DNA material in limited such as liquid biopsy (Rossi and Zamarchi, 2019). Circulating cancer cells (CTCs) and circulating tumor DNA (ctDNA) are present in the bodily fluids of cancer patients and can serve as diagnostic markers. Epigenomic markers in liquid biopsy have recently been investigated and been proven very informative for diagnosis (Liu et al., 2020). Due to low level of genetic material from tumors in bodily fluids, sensitive methods are key to increasing detection accuracy. Single-cell transcriptomic assays have already been utilized in liquid biopsy studies (Lim et al., 2019). Extending the applications of sc-epigenomic methods to CTCs can thus be instrumental in advancing our understanding of the epigenomic layers and their biomarker potentials by liquid biopsy. Finally, advancements in spatial epigenomics can add a new dimension to the cell-to-cell regulatory relationships in the context of tissues in the future. Single cell technologies, though rapidly advancing, have yet to become comparable with bulk epigenomic assays in terms of optimization and standardization of both the experimental and data analysis practices. Therefore, depending on the purpose of a study, it may not always be preferential to perform an assay at the single-cell level and one has to carefully weigh the advantages and disadvantages depending on the context. The additional experimental complications and increased noise in the data are worth dealing with when tissue composition, cell type heterogeneity, and cell-cell interactions are the crucial goals of a research. Furthermore, in contexts such as studying rare cell populations e.g., metastatic cells in liquid biopsy, or few-cell stages during early development, the low number of cells of interest make it impossible to use bulk approaches and thus single-cell assays become the method of choice. Finally, single-cell multi-omic assays allow for the assessment of the complicated regulatory relationships between various levels of the epigenome at an accuracy and resolution level not possible with bulk multi-omics approaches. Therefore, re-evaluation of these relationships now using single-cell datasets is expected to provide new functional insights in the field.

## Author Contributions

MM sketched the overall structure and content of the review. All authors contributed to writing the manuscript and preparing the tables. All authors read and approved the final manuscript. MS made the figures. MM revised and edited the final manuscript.

## Conflict of Interest

The authors declare that the research was conducted in the absence of any commercial or financial relationships that could be construed as a potential conflict of interest.

## References

[B1] Allis C. D. Jenuwein T. (2016). The molecular hallmarks of epigenetic control. *Nat. Rev. Genet.* 17 487–500. 10.1038/nrg.2016.59 27346641

[B2] Angermueller C. Clark S. J. Lee H. J. Macaulay I. C. Teng M. J. Hu T. X. (2016). Parallel single-cell sequencing links transcriptional and epigenetic heterogeneity. *Nat. Methods* 13 229–232. 10.1038/nmeth.3728 26752769 PMC4770512

[B3] Aughey G. N. Cheetham S. W. Southall T. D. (2019). DamID as a versatile tool for understanding gene regulation. *Development* 146:dev173666. 30877125 10.1242/dev.173666 PMC6451315

[B4] Barnett K. R. Decato B. E. Scott T. J. Hansen T. J. Chen B. Attalla J. (2020). ATAC-Me captures prolonged DNA methylation of dynamic chromatin accessibility loci during cell fate Transitions. *Mol. Cell* 77 1350–1364.e6. 31999955 10.1016/j.molcel.2020.01.004 PMC7169048

[B5] Bartosovic M. Kabbe M. Castelo-Branco G. (2021). Single-cell CUT&Tag profiles histone modifications and transcription factors in complex tissues. *Nat. Biotechnol.* [Epub ahead of print]. 10.1038/s41587-021-00869-9 PMC7611252 33846645

[B6] Booth M. J. Branco M. R. Ficz G. Oxley D. Krueger F. Reik W. (2012). Quantitative sequencing of 5-methylcytosine and 5-hydroxymethylcytosine at single-base resolution. *Science* 336 934–937. 10.1126/science.1220671 22539555

[B7] Boyle A. P. Davis S. Shulha H. P. Meltzer P. Margulies E. H. Weng Z. (2008). High-resolution mapping and characterization of open chromatin across the genome. *Cell* 132 311–322. 10.1016/j.cell.2007.12.014 18243105 PMC2669738

[B8] Buenrostro J. D. Wu B. Chang H. Y. Greenleaf W. J. (2015a). ATAC-seq: a method for assaying chromatin accessibility genome-wide. *Curr. Protoc. Mol. Biol.* 109 21.29.1–21.29.9. 10.1002/0471142727.mb2129s109 PMC4374986 25559105

[B9] Buenrostro J. D. Wu B. Litzenburger U. M. Ruff D. Gonzales M. L. Snyder M. P. (2015b). Single-cell chromatin accessibility reveals principles of regulatory variation. *Nature* 523 486–490. 10.1038/nature14590 26083756 PMC4685948

[B10] Cao J. Cusanovich D. A. Ramani V. Aghamirzaie D. Pliner H. A. Hill A. J. (2018). Joint profiling of chromatin accessibility and gene expression in thousands of single cells. *Science* 361 1380–1385. 10.1126/science.aau0730 30166440 PMC6571013

[B11] Carter B. Ku W. L. Kang J. Y. Hu G. Perrie J. Tang Q. (2019). Mapping histone modifications in low cell number and single cells using antibody-guided chromatin tagmentation (ACT-seq). *Nat. Commun.* 10:3747. 31431618 10.1038/s41467-019-11559-1 PMC6702168

[B12] Chang P. Gohain M. Yen M. R. Chen P. Y. (2018). Computational methods for assessing chromatin hierarchy. *Comput. Struct. Biotechnol. J.* 16 43–53. 10.1016/j.csbj.2018.02.003 29686798 PMC5910504

[B13] Clark S. J. Argelaguet R. Kapourani C. A. Stubbs T. M. Lee H. J. Alda-Catalinas C. (2018). scNMT-seq enables joint profiling of chromatin accessibility DNA methylation and transcription in single cells. *Nat. Commun.* 9:781. 29472610 10.1038/s41467-018-03149-4 PMC5823944

[B14] Clark S. J. Harrison J. Paul C. L. Frommer M. (1994). High sensitivity mapping of methylated cytosines. *Nucleic Acids Res.* 22 2990–2997. 10.1093/nar/22.15.2990 8065911 PMC310266

[B15] Clark S. J. Statham A. Stirzaker C. Molloy P. L. Frommer M. (2006). DNA methylation: bisulphite modification and analysis. *Nat. Protoc.* 1 2353–2364. 10.1038/nprot.2006.324 17406479

[B16] Cusanovich D. A. Daza R. Adey A. Pliner H. A. Christiansen L. Gunderson K. L. (2015). Multiplex single cell profiling of chromatin accessibility by combinatorial cellular indexing. *Science* 348 910–914. 10.1126/science.aab1601 25953818 PMC4836442

[B17] de Luca K. L. Kind J. (2021). Single-cell DamID to capture contacts between DNA and the nuclear lamina in individual mammalian cells. *Methods Mol. Biol.* 2157 159–172. 10.1007/978-1-0716-0664-3_9 32820403

[B18] Dean F. B. Hosono S. Fang L. Wu X. Faruqi A. F. Bray-Ward P. (2002). Comprehensive human genome amplification using multiple displacement amplification. *Proc. Natl. Acad. Sci. U.S.A.* 99 5261–5266. 10.1073/pnas.082089499 11959976 PMC122757

[B19] DeAngelis J. T. Farrington W. J. Tollefsbol T. O. (2008). An overview of epigenetic assays. *Mol. Biotechnol.* 38 179–183. 10.1007/s12033-007-9010-y 17943463 PMC2423347

[B20] Deng Y. Zhang D. Liu Y. Su G. Enninful A. Bai Z. (2021). Spatial epigenome sequencing at tissue scale and cellular level. *bioaRxiv* [Preprint]. 10.1101/2021.03.11.434985

[B21] Diep D. Plongthongkum N. Gore A. Fung H. L. Shoemaker R. Zhang K. (2012). Library-free methylation sequencing with bisulfite padlock probes. *Nat. Methods* 9 270–272. 10.1038/nmeth.1871 22306810 PMC3461232

[B22] Estecio M. R. Yan P. S. Huang T. H. Issa J. P. (2008). Methylated CpG Island Amplification and Microarray (MCAM) for high-throughput analysis of DNA methylation. *CSH Protoc.* 2008:db.rot4974. 10.1101/pdb.prot4974 21356790

[B23] Fang R. Yu M. Li G. Chee S. Liu T. Schmitt A. D. (2016). Mapping of long-range chromatin interactions by proximity ligation-assisted ChIP-seq. *Cell Res.* 26 1345–1348. 10.1038/cr.2016.137 27886167 PMC5143423

[B24] Flusberg B. A. Webster D. R. Lee J. H. Travers K. J. Olivares E. C. Clark T. A. (2010). Direct detection of DNA methylation during single-molecule, real-time sequencing. *Nat. Methods* 7 461–465. 10.1038/nmeth.1459 20453866 PMC2879396

[B25] Fullwood M. J. Liu M. H. Pan Y. F. Liu J. Xu H. Mohamed Y. B. (2009). An oestrogen-receptor-alpha-bound human chromatin interactome. *Nature* 462 58–64. 19890323 10.1038/nature08497 PMC2774924

[B26] Giresi P. G. Kim J. McDaniell R. M. Iyer V. R. Lieb J. D. (2007). FAIRE (Formaldehyde-Assisted Isolation of Regulatory Elements) isolates active regulatory elements from human chromatin. *Genome Res.* 17 877–885. 10.1101/gr.5533506 17179217 PMC1891346

[B27] Gontarz P. Fu S. Xing X. Liu S. Miao B. Bazylianska V. (2020). Comparison of differential accessibility analysis strategies for ATAC-seq data. *Sci. Rep.* 10:10150. 32576878 10.1038/s41598-020-66998-4 PMC7311460

[B28] Greil F. Moorman C. van Steensel B. (2006). DamID: mapping of in vivo protein-genome interactions using tethered DNA adenine methyltransferase. *Methods Enzymol.* 410 342–359. 10.1016/s0076-6879(06)10016-6 16938559

[B29] Gu C. Liu S. Wu Q. Zhang L. Guo F. (2019). Integrative single-cell analysis of transcriptome, DNA methylome and chromatin accessibility in mouse oocytes. *Cell Res.* 29 110–123. 10.1038/s41422-018-0125-4 30560925 PMC6355938

[B30] Guo F. Li L. Li J. Wu X. Hu B. Zhu P. (2017). Single-cell multi-omics sequencing of mouse early embryos and embryonic stem cells. *Cell Res.* 27 967–988. 10.1038/cr.2017.82 28621329 PMC5539349

[B31] Guo H. Zhu P. Wu X. Li X. Wen L. Tang F. (2013). Single-cell methylome landscapes of mouse embryonic stem cells and early embryos analyzed using reduced representation bisulfite sequencing. *Genome Res.* 23 2126–2135. 10.1101/gr.161679.113 24179143 PMC3847781

[B32] Han L. Wu H. J. Zhu H. Kim K. Y. Marjani S. L. Riester M. (2017). Bisulfite-independent analysis of CpG island methylation enables genome-scale stratification of single cells. *Nucleic Acids Res.* 45:e77. 28126923 10.1093/nar/gkx026 PMC5605247

[B33] Harada A. Maehara K. Handa T. Arimura Y. Nogami J. Hayashi-Takanaka Y. (2019). A chromatin integration labelling method enables epigenomic profiling with lower input. *Nat.Cell Biol.* 21 287–296. 10.1038/s41556-018-0248-3 30532068

[B34] Harris R. A. Wang T. Coarfa C. Nagarajan R. P. Hong C. Downey S. L. (2010). Comparison of sequencing-based methods to profile DNA methylation and identification of monoallelic epigenetic modifications. *Nat. Biotechnol.* 28 1097–1105. 20852635 10.1038/nbt.1682 PMC2955169

[B35] Hayatsu H. (2008). Discovery of bisulfite-mediated cytosine conversion to uracil, the key reaction for DNA methylation analysis–a personal account. *Proc. Jpn. Acad. Ser. B Phys. Biol. Sci.* 84 321–330. 10.2183/pjab.84.321 18941305 PMC3722019

[B36] He Q. Johnston J. Zeitlinger J. (2015). ChIP-nexus enables improved detection of in vivo transcription factor binding footprints. *Nat. Biotechnol.* 33 395–401. 10.1038/nbt.3121 25751057 PMC4390430

[B37] Heiss J. A. Brennan K. J. Baccarelli A. A. Téllez-Rojo M. M. Estrada-Gutiérrez G. Wright R. O. (2020). Battle of epigenetic proportions: comparing Illumina’s EPIC methylation microarrays and TruSeq targeted bisulfite sequencing. *Epigenetics* 15 174–182. 10.1080/15592294.2019.1656159 31538540 PMC6961683

[B38] Hou Y. Guo H. Cao C. Li X. Hu B. Zhu P. (2016). Single-cell triple omics sequencing reveals genetic, epigenetic, and transcriptomic heterogeneity in hepatocellular carcinomas. *Cell Res.* 26 304–319. 10.1038/cr.2016.23 26902283 PMC4783472

[B39] Hu P. Zhang W. Xin H. Deng G. (2016). Single cell isolation and analysis. *Front. Cell. Dev. Biol.* 4:116. 10.3389/fcell.2016.00116 27826548 PMC5078503

[B40] Hu Y. An Q. Guo Y. Zhong J. Fan S. Rao P. (2019). Simultaneous profiling of mRNA transcriptome and DNA methylome from a single cell. *Methods Mol. Biol.* 1979 363–377. 10.1007/978-1-4939-9240-9_21 31028648

[B41] Hu Y. Huang K. An Q. Du G. Hu G. Xue J. (2016). Simultaneous profiling of transcriptome and DNA methylome from a single cell. *Genome Biol.* 17:88. 27150361 10.1186/s13059-016-0950-z PMC4858893

[B42] Irizarry R. A. Ladd-Acosta C. Carvalho B. Wu H. Brandenburg S. A. Jeddeloh J. A. (2008). Comprehensive high-throughput arrays for relative methylation (CHARM). *Genome Res.* 18 780–790. 10.1101/gr.7301508 18316654 PMC2336799

[B43] Jin W. Tang Q. Wan M. Cui K. Zhang Y. Ren G. (2015). Genome-wide detection of DNase I hypersensitive sites in single cells and FFPE tissue samples. *Nature* 528 142–146. 10.1038/nature15740 26605532 PMC4697938

[B44] Jung M. Kadam S. Xiong W. Rauch T. A. Jin S. G. Pfeifer G. P. (2015). MIRA-seq for DNA methylation analysis of CpG islands. *Epigenomics* 7 695–706. 10.2217/epi.15.33 25881900 PMC4607651

[B45] Kacmarczyk T. J. Fall M. P. Zhang X. Xin Y. Li Y. Alonso A. (2018). “Same difference”: comprehensive evaluation of four DNA methylation measurement platforms. *Epigenetics Chromatin* 11:21. 29801521 10.1186/s13072-018-0190-4 PMC5970534

[B46] Kaya-Okur H. S. Wu S. J. Codomo C. A. Pledger E. S. Bryson T. D. Henikoff J. G. (2019). CUT&Tag for efficient epigenomic profiling of small samples and single cells. *Nat. Commun.* 10:1930. 31036827 10.1038/s41467-019-09982-5 PMC6488672

[B47] Kelly T. K. Liu Y. Lay F. D. Liang G. Berman B. P. Jones P. A. (2012). Genome-wide mapping of nucleosome positioning and DNA methylation within individual DNA molecules. *Genome Res.* 22 2497–2506. 10.1101/gr.143008.112 22960375 PMC3514679

[B48] Kelsey G. Stegle O. Reik W. (2017). Single-cell epigenomics: recording the past and predicting the future. *Science* 358 69–75. 10.1126/science.aan6826 28983045

[B49] Khulan B. Thompson R. F. Ye K. Fazzari M. J. Suzuki M. Stasiek E. (2006). Comparative isoschizomer profiling of cytosine methylation: the HELP assay. *Genome Res.* 16 1046–1055. 10.1101/gr.5273806 16809668 PMC1524864

[B50] Kind J. Pagie L. de Vries SS Nahidiazar L. Dey S. S. Bienko M. (2015). Genome-wide maps of nuclear lamina interactions in single human cells. *Cell* 163 134–147. 10.1016/j.cell.2015.08.040 26365489 PMC4583798

[B51] Klemm S. L. Shipony Z. Greenleaf W. J. (2019). Chromatin accessibility and the regulatory epigenome. *Nat. Rev. Genet.* 20 207–220. 10.1038/s41576-018-0089-8 30675018

[B52] Ku W. L. Nakamura K. Gao W. Cui K. Hu G. Tang Q. (2019). Single-cell chromatin immunocleavage sequencing (scChIC-seq) to profile histone modification. *Nat. Methods* 16 323–325. 10.1038/s41592-019-0361-7 30923384 PMC7187538

[B53] Larsson L. Frisen J. Lundeberg J. (2021). Spatially resolved transcriptomics adds a new dimension to genomics. *Nat. Methods* 18 15–18. 10.1038/s41592-020-01038-7 33408402

[B54] Lee D. S. Luo C. Zhou J. Chandran S. Rivkin A. Bartlett A. (2019). Simultaneous profiling of 3D genome structure and DNA methylation in single human cells. *Nat. Methods* 16 999–1006. 10.1038/s41592-019-0547-z 31501549 PMC6765423

[B55] Lhoumaud P. Sethia G. Izzo F. Sakellaropoulos T. Snetkova V. Vidal S. (2019). EpiMethylTag: simultaneous detection of ATAC-seq or ChIP-seq signals with DNA methylation. *Genome Biol.* 20:248. 31752933 10.1186/s13059-019-1853-6 PMC6868874

[B56] Li G. Liu Y. Zhang Y. Kubo N. Yu M. Fang R. (2019). Joint profiling of DNA methylation and chromatin architecture in single cells. *Nat. Methods* 16 991–993. 10.1038/s41592-019-0502-z 31384045 PMC6765429

[B57] Lim S. B. Di Lee W. Vasudevan J. Lim W. T. Lim C. T. (2019). Liquid biopsy: one cell at a time. *NPJ Precis. Oncol.* 3:23. 31602399 10.1038/s41698-019-0095-0 PMC6775080

[B58] Liu L. Liu C. Quintero A. Wu L. Yuan Y. Wang M. (2019). Deconvolution of single-cell multi-omics layers reveals regulatory heterogeneity. *Nat. Commun.* 10:470. 30692544 10.1038/s41467-018-08205-7 PMC6349937

[B59] Liu M. C. Oxnard G. R. Klein E. A. Swanton C. Seiden M. V. Ccga Consortium. (2020). Sensitive and specific multi-cancer detection and localization using methylation signatures in cell-free DNA. *Ann. Oncol.* 31 745–759. 33506766 10.1016/j.annonc.2020.02.011 PMC8274402

[B60] Lo P. K. Zhou Q. (2018). Emerging techniques in single-cell epigenomics and their applications to cancer research. *J. Clin. Genom.* 1:10.4172/JCG.1000103 30079405 PMC6070152

[B61] Meissner A. Gnirke A. Bell G. W. Ramsahoye B. Lander E. S. Jaenisch R. (2005). Reduced representation bisulfite sequencing for comparative high-resolution DNA methylation analysis. *Nucleic Acids Res.* 33 5868–5877. 10.1093/nar/gki901 16224102 PMC1258174

[B62] Milne T. A. Zhao K. Hess J. L. (2009). Chromatin immunoprecipitation (ChIP) for analysis of histone modifications and chromatin-associated proteins. *Methods Mol. Biol.* 538 409–423. 10.1007/978-1-59745-418-6_21 19277579 PMC4157307

[B63] Miura F. Enomoto Y. Dairiki R. Ito T. (2012). Amplification-free whole-genome bisulfite sequencing by post-bisulfite adaptor tagging. *Nucleic Acids Res.* 40:e136. 10.1093/nar/gks454 22649061 PMC3458524

[B64] Mulqueen R. M. Pokholok D. Norberg S. J. Torkenczy K. A. Fields A. J. Sun D. (2018). Highly scalable generation of DNA methylation profiles in single cells. *Nat. Biotechnol.* 36 428–431. 10.1038/nbt.4112 29644997 PMC5938134

[B65] Mumbach M. R. Rubin A. J. Flynn R. A. Dai C. Khavari P. A. Greenleaf W. J. (2016). HiChIP: efficient and sensitive analysis of protein-directed genome architecture. *Nat. Methods* 13 919–922. 10.1038/nmeth.3999 27643841 PMC5501173

[B66] Nagano T. Lubling Y. Stevens T. J. Schoenfelder S. Yaffe E. Dean W. (2013). Single-cell Hi-C reveals cell-to-cell variability in chromosome structure. *Nature* 502 59–64. 10.1038/nature12593 24067610 PMC3869051

[B67] Oakes C. C. La Salle S. Robaire B. Trasler J. M. (2006). Evaluation of a quantitative DNA methylation analysis technique using methylation-sensitive/dependent restriction enzymes and real-time PCR. *Epigenetics* 1 146–152. 10.4161/epi.1.3.3392 17965615

[B68] Oda M. Glass J. L. Thompson R. F. Mo Y. Olivier E. N. Figueroa M. E. (2009). High-resolution genome-wide cytosine methylation profiling with simultaneous copy number analysis and optimization for limited cell numbers. *Nucleic Acids Res.* 37 3829–3839. 10.1093/nar/gkp260 19386619 PMC2709560

[B69] Orchard P. Kyono Y. Hensley J. Kitzman J. O. Parker S. C. J. (2020). Quantification, dynamic visualization, and validation of bias in ATAC-Seq data with ataqv. *Cell Syst.* 10 298–306.e4. 32213349 10.1016/j.cels.2020.02.009 PMC8245295

[B70] Park P. J. (2009). ChIP-seq: advantages and challenges of a maturing technology. *Nat. Rev. Genet.* 10 669–680. 10.1038/nrg2641 19736561 PMC3191340

[B71] Pelizzola M. Koga Y. Urban A. E. Krauthammer M. Weissman S. Halaban R. (2008). MEDME: an experimental and analytical methodology for the estimation of DNA methylation levels based on microarray derived MeDIP-enrichment. *Genome Res.* 18 1652–1659. 10.1101/gr.080721.108 18765822 PMC2556264

[B72] Pott S. (2017). Simultaneous measurement of chromatin accessibility, DNA methylation, and nucleosome phasing in single cells. *Elife* 6:e23203. 28653622 10.7554/eLife.23203 PMC5487215

[B73] Ramani V. Deng X. Qiu R. Gunderson K. L. Steemers F. J. Disteche C. M. (2017). Massively multiplex single-cell Hi-C. *Nat. Methods* 14 263–266. 10.1038/nmeth.4155 28135255 PMC5330809

[B74] Rauch T. A. Wu X. Zhong X. Riggs A. D. Pfeifer G. P. (2009). A human B cell methylome at 100-base pair resolution. *Proc. Natl. Acad. Sci. U.S.A.* 106 671–678. 10.1073/pnas.0812399106 19139413 PMC2621253

[B75] Rhee H. S. Pugh B. F. (2012). ChIP-exo method for identifying genomic location of DNA-binding proteins with near-single-nucleotide accuracy. *Curr. Protoc. Mol. Biol.* Chapter 21:Unit21.24. 10.1002/0471142727.mb2124s100 PMC3813302 23026909

[B76] Rivera C. M. Ren B. (2013). Mapping human epigenomes. *Cell* 155 39–55. 10.1016/j.cell.2013.09.011 24074860 PMC3838898

[B77] Rossi E. Zamarchi R. (2019). Single-cell analysis of circulating tumor cells: how far have we come in the -omics era? *Front. Genet.* 10:958. 10.3389/fgene.2019.00958 31681412 PMC6811661

[B78] Rotem A. Ram O. Shoresh N. Sperling R. A. Goren A. Weitz D. A. (2015). Single-cell ChIP-seq reveals cell subpopulations defined by chromatin state. *Nat. Biotechnol.* 33 1165–1172. 10.1038/nbt.3383 26458175 PMC4636926

[B79] Schmidl C. Rendeiro A. F. Sheffield N. C. Bock C. (2015). ChIPmentation: fast, robust, low-input ChIP-seq for histones and transcription factors. *Nat. Methods* 12 963–965. 10.1038/nmeth.3542 26280331 PMC4589892

[B80] Schones D. E. Cui K. Cuddapah S. Roh T. Y. Barski A. Wang Z. (2008). Dynamic regulation of nucleosome positioning in the human genome. *Cell* 132 887–898. 10.1016/j.cell.2008.02.022 18329373 PMC10894452

[B81] Simpson J. T. Workman R. E. Zuzarte P. C. David M. Dursi L. J. Timp W. (2017). Detecting DNA cytosine methylation using nanopore sequencing. *Nat. Methods* 14 407–410. 10.1038/nmeth.4184 28218898

[B82] Singer B. D. (2019). A practical guide to the measurement and analysis of DNA methylation. *Am. J. Respir. Cell Mol. Biol.* 61 417–428. 10.1165/rcmb.2019-0150tr 31264905 PMC6775954

[B83] Skene P. J. Henikoff S. (2017). An efficient targeted nuclease strategy for high-resolution mapping of DNA binding sites. *Elife* 6:e21856. 28079019 10.7554/eLife.21856 PMC5310842

[B84] Small E. C. Xi L. Wang J. P. Widom J. Licht J. D. (2014). Single-cell nucleosome mapping reveals the molecular basis of gene expression heterogeneity. *Proc. Natl. Acad. Sci. U.S.A.* 111 E2462–E2471. 24889621 10.1073/pnas.1400517111 PMC4066511

[B85] Smallwood S. A. Lee H. J. Angermueller C. Krueger F. Saadeh H. Peat J. (2014). Single-cell genome-wide bisulfite sequencing for assessing epigenetic heterogeneity. *Nat. Methods* 11 817–820. 10.1038/nmeth.3035 25042786 PMC4117646

[B86] Smith A. R. Smith R. G. Pishva E. Hannon E. Roubroeks J. A. Y. Burrage J. (2019). Parallel profiling of DNA methylation and hydroxymethylation highlights neuropathology-associated epigenetic variation in Alzheimer’s disease. *Clin. Epigenet.* 11:52. 10.1186/s13148-019-0636-y PMC6429761 30898171

[B87] Song L. Crawford G. E. (2010). DNase-seq: a high-resolution technique for mapping active gene regulatory elements across the genome from mammalian cells. *Cold Spring Harb. Protoc.* 2010:pdb.prot5384. 20150147 10.1101/pdb.prot5384 PMC3627383

[B88] Stahl P. L. Salmén F. Vickovic S. Lundmark A. Navarro J. F. Magnusson J. (2016). Visualization and analysis of gene expression in tissue sections by spatial transcriptomics. *Science* 353 78–82. 10.1126/science.aaf2403 27365449

[B89] Sun W. Zang L. Shu Q. Li X. (2014). From development to diseases: the role of 5hmC in brain. *Genomics* 104 347–351. 10.1016/j.ygeno.2014.08.021 25205306

[B90] Sun Z. Terragni J. Borgaro J. G. Liu Y. Yu L. Guan S. (2013). High-resolution enzymatic mapping of genomic 5-hydroxymethylcytosine in mouse embryonic stem cells. *Cell Rep.* 3 567–576. 10.1016/j.celrep.2013.01.001 23352666 PMC3743234

[B91] Swanson E. Lord C. Reading J. Heubeck A. T. Genge P. C. Thomson Z. (2021). Simultaneous trimodal single-cell measurement of transcripts, epitopes, and chromatin accessibility using TEA-seq. *Elife* 10:e63632. 33835024 10.7554/eLife.63632 PMC8034981

[B92] Tan L. Xiong L. Xu W. Wu F. Huang N. Xu Y. (2013). Genome-wide comparison of DNA hydroxymethylation in mouse embryonic stem cells and neural progenitor cells by a new comparative hMeDIP-seq method. *Nucleic Acids Res.* 41:e84. 10.1093/nar/gkt091 23408859 PMC3627583

[B93] Tanić M. Moghul I. Rodney S. Dhami P. Vaikkinen H. Ambrose J. (2021). Performance comparison and in-silico harmonisation of commercial platforms for DNA methylome analysis by targeted bisulfite sequencing. *bioaRxiv* [Preprint]. 10.1101/2021.03.12.435105 35654977

[B94] Tse O. Y. O. Jiang P. Cheng S. H. Peng W. Shang H. Wong J. (2021). Genome-wide detection of cytosine methylation by single molecule real-time sequencing. *Proc. Natl. Acad. Sci. U.S.A.* 118:e2019768118. 10.1073/pnas.2019768118 33495335 PMC7865158

[B95] van Berkum N. L. Lieberman-Aiden E. Williams L. Imakaev M. Gnirke A. Mirny L. A. (2010). Hi-C: a method to study the three-dimensional architecture of genomes. *J. Vis. Exp.* 39:1869. 10.3791/1869 PMC3149993 20461051

[B96] van Steensel B. Belmont A. S. (2017). Lamina-associated domains: links with chromosome architecture, heterochromatin, and gene repression. *Cell* 169 780–791. 10.1016/j.cell.2017.04.022 28525751 PMC5532494

[B97] Wagner J. R. Busche S. Ge B. Kwan T. Pastinen T. Blanchette M. (2014). The relationship between DNA methylation, genetic and expression inter-individual variation in untransformed human fibroblasts. *Genome Biol.* 15:R37. 24555846 10.1186/gb-2014-15-2-r37 PMC4053980

[B98] Wang K. C. Chang H. Y. (2018). Epigenomics: technologies and applications. *Circ. Res.* 122 1191–1199. 10.1161/circresaha.118.310998 29700067 PMC5929475

[B99] Wang Q. Xiong H. Xiong H. Yu X. Liu Y. Zhang J. (2019). CoBATCH for high-throughput single-cell epigenomic profiling. *Mol. Cell* 76 206–216.e7. 10.1016/j.molcel.2019.07.015 31471188

[B100] Weber M. Davies J. J. Wittig D. Oakeley E. J. Haase M. Lam W. L. (2005). Chromosome-wide and promoter-specific analyses identify sites of differential DNA methylation in normal and transformed human cells. *Nat. Genet.* 37 853–862. 10.1038/ng1598 16007088

[B101] Worm Orntoft M. B. Jensen S. O. Hansen T. B. Bramsen J. B. Andersen C. L. (2017). Comparative analysis of 12 different kits for bisulfite conversion of circulating cell-free DNA. *Epigenetics* 12 626–636. 10.1080/15592294.2017.1334024 28557629 PMC5687322

[B102] Wu F. Olson B. G. Yao J. (2016). DamID-seq: genome-wide mapping of protein-DNA interactions by high throughput sequencing of adenine-methylated DNA fragments. *J. Vis. Exp.* 107:e53620. 10.3791/53620 PMC4781701 26862720

[B103] Xiao C. L. Zhu S. He M. Chen D. Zhang Q. Chen Y. (2018). N(6)-methyladenine DNA modification in the human genome. *Mol. Cell* 71 306–318.e7. 30017583 10.1016/j.molcel.2018.06.015

[B104] Yan P. S. Potter D. Deatherage D. E. Huang T. H. Lin S. (2009). Differential methylation hybridization: profiling DNA methylation with a high-density CpG island microarray. *Methods Mol. Biol.* 507 89–106. 10.1007/978-1-59745-522-0_8 18987809

[B105] Yang Y. Scott S. A. (2017). DNA methylation profiling using long-read single molecule real-time bisulfite sequencing (SMRT-BS). *Methods Mol. Biol.* 1654 125–134. 10.1007/978-1-4939-7231-9_8 28986786

[B106] Yu M. Hon G. C. Szulwach K. E. Song C. X. Zhang L. Kim A. (2012). Base-resolution analysis of 5-hydroxymethylcytosine in the mammalian genome. *Cell* 149 1368–1380. 10.1016/j.cell.2012.04.027 22608086 PMC3589129

[B107] Zhu C. Gao Y. Guo H. Xia B. Song J. Wu X. (2017). Single-Cell 5-formylcytosine landscapes of mammalian early embryos and ESCs at single-base resolution. *Cell Stem Cell* 20 720–731.e5. 28343982 10.1016/j.stem.2017.02.013

[B108] Zhu C. Preissl S. Ren B. (2020). Single-cell multimodal omics: the power of many. *Nat. Methods* 17 11–14. 10.1038/s41592-019-0691-5 31907462

[B109] Zhu C. Yu M. Huang H. Juric I. Abnousi A. Hu R. (2019). An ultra high-throughput method for single-cell joint analysis of open chromatin and transcriptome. *Nat. Struct. Mol. Biol.* 26 1063–1070. 10.1038/s41594-019-0323-x 31695190 PMC7231560

